# A new iterative initialization of EM algorithm for Gaussian mixture models

**DOI:** 10.1371/journal.pone.0284114

**Published:** 2023-04-13

**Authors:** Jie You, Zhaoxuan Li, Junli Du

**Affiliations:** College of Science, Northwest A&F University, Yangling, Shaanxi, P.R. China; TU Wien: Technische Universitat Wien, AUSTRIA

## Abstract

**Background:**

The expectation maximization (EM) algorithm is a common tool for estimating the parameters of Gaussian mixture models (GMM). However, it is highly sensitive to initial value and easily gets trapped in a local optimum.

**Method:**

To address these problems, a new iterative method of EM initialization (MRIPEM) is proposed in this paper. It incorporates the ideas of multiple restarts, iterations and clustering. In particular, the mean vector and covariance matrix of sample are calculated as the initial values of the iteration. Then, the optimal feature vector is selected from the candidate feature vectors by the maximum Mahalanobis distance as a new partition vector for clustering. The parameter values are renewed continuously according to the clustering results.

**Results:**

To verify the applicability of the MRIPEM, we compared it with other two popular initialization methods on simulated and real datasets, respectively. The comparison results of the three stochastic algorithms indicate that MRIPEM algorithm is comparable in relatively high dimensions and high overlaps and significantly better in low dimensions and low overlaps.

## 1 Introduction

**Fig 1:**
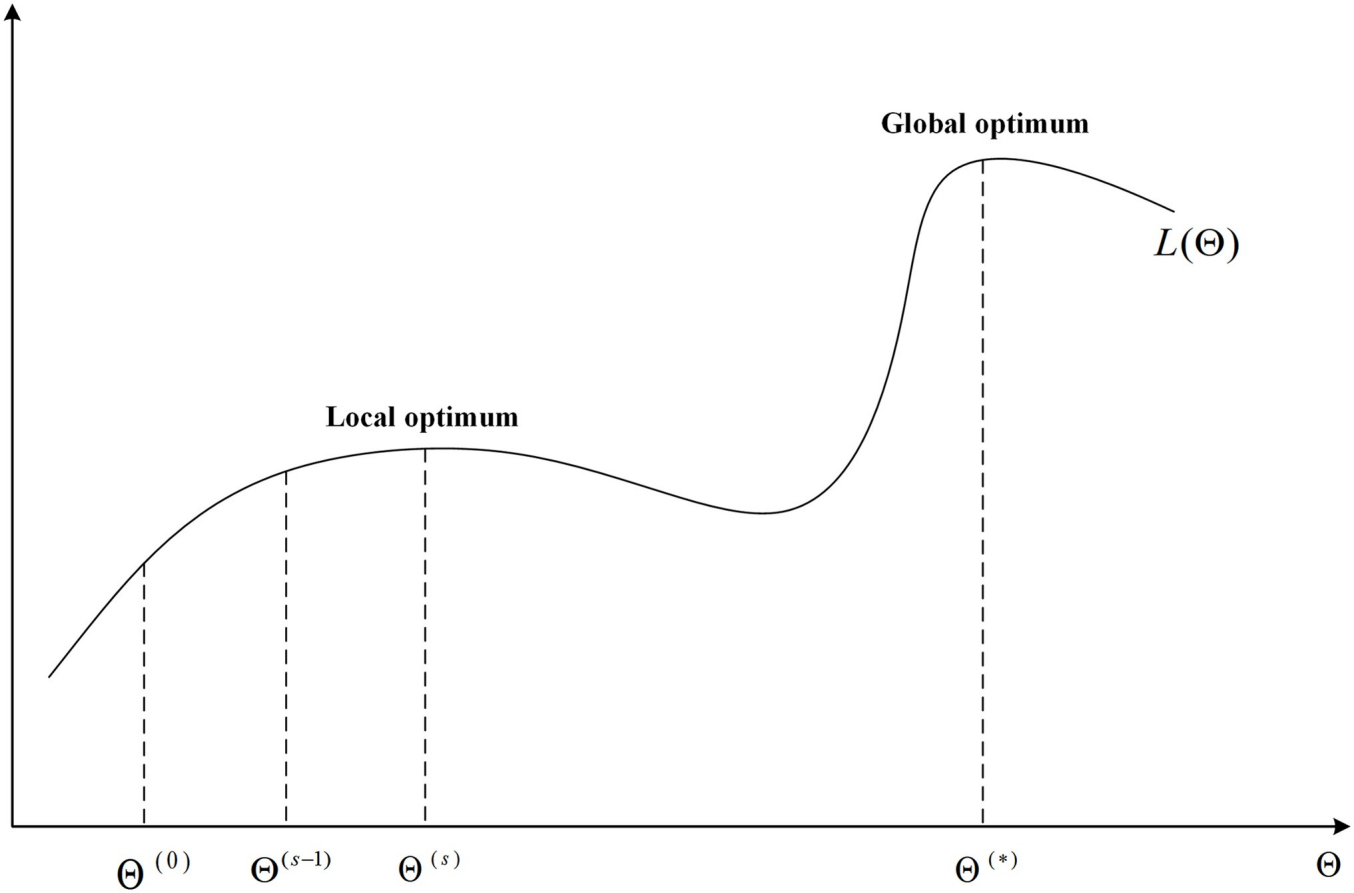
Relationship curve between *L*(Θ) and Θ. Θ: the initial parameter of the EM algorithm; *L*(Θ): the likelihood function about Θ.

Gaussian mixture model (GMM) is a very useful tool, which is widely used in complex probability distribution modeling, such as data classification [1], image classification and segmentation [2–4], speech recognition [5], etc. The Gaussian mixture model is composed of *K* single Gaussian distributions. For a single Gaussian distribution, the parameters are usually estimated by using the maximum likelihood estimation (MLE) method, but this is not applicable to GMM. In fact, it is not known in advance which component each observation belongs to in GMM due to introduce the hidden variables. The expectation-maximization method (EM), introduced by Dempster et al. [6], can complete the parameter estimation of the GMM by iteratively constructing the lower limit of the likelihood function to continuously improve the value of the likelihood function. However, this procedure is highly sensitive to initialization and easily gets trapped in a local optimum [7, 8]. As shown in Fig 1, when the likelihood function is nonconvex, the EM algorithm may terminate the iteration before reaching the global optimum. Thus, initialization research is very necessary for EM algorithm.

At present, many initialization methods of the EM algorithm for GMM have been proposed, which are mainly divided into two situations with a known and an unknown numbers of components *K*. When the number of components *K* is known, some initialization methods belong to the deterministic strategy. For example, the initialization method in the document [9] is based on hierarchical agglomerative clustering (HAC), which uses Ward’s criterion measurement to obtain the means of the initial model. The procedure proposed by Maitra relies on detecting the best local modes [10]. The deterministic methods may lead to an incorrect solution or even no solution when the likelihood function is unbounded [11]. In comparison, stochastic initialization strategies can bring improvements with the increase of runs. The standard program of stochastic initialization is the multiple restart method (MREM) [12]. In this approach, the EM algorithm is run many times with different random initial conditions. The parameters corresponding to the highest log likelihood are returned as the final parameter estimation. When the sample size is large, the method is still easy to fall into local optimization. The emEM [13] and RndEM [10] algorithms are the other two typical representatives of stochastic initialization strategies. The emEM algorithm starts with the phase called short EM at which the EM algorithm iterates multiple times from different random points according to a lax convergence criterion. The set of parameters that produced the highest likelihood value is then used for the final long EM run. RndEM is a variant of emEM algorithm. It involves the same stages as emEM, but the short EM phase stops after the very first parameter estimation. In the paper of Blömer and Bujna [14], two new initialization methods are presented based on the well-known K-means++ algorithm and the Gonzalez algorithm. In the method of rnd-maxmin [15], the initialization mean is selected from the random subset of candidate eigenvectors by application of Mahalanobis distance. The covariance matrix of the component is initialized by randomly generating the eigenvalues and eigenvectors.

The above approaches generally need to give the number of components *K* in advance. In fact, if *K* is unknown, it can be assumed firstly to belong to a set of values K. For each different K∈K, the initialization method and EM algorithm are run. The *K* value of best fitting model is to be chosen based on some test procedures or criteria (usually log-likelihood value). Moreover, many initialization methods integrate the estimation of *K* in the process of calculating parameters. A clustering algorithm using fast search of density peak points (DPC) [16] can predict the number of components *K* and centers of the initial class. The method of *Σ*-EM [17] initializes the mean vectors by choosing points that have a higher concentration of neighbors and the covariance matrix by using a truncated normal distribution. In the paper of Verbeek et al. [18], a greedy algorithm is presented which does not need an initialization but needs to construct a whole sequence of mixture models with *m* = 1, …, *K* components instead. Subsequently, the optimized greedy initialization methods are proposed [19, 20]. Another method is to apply Rough-Enhanced-Bayes mixture estimation (REBMIX) to the initialization of the EM algorithm [21]. In fact, this approach combines a new type of clustering algorithm with the EM algorithm to improve the accuracy of the results, and the approach itself is not innovative.

Clearly, there is no “the best initialization algorithm” that can be applied to all instances. The performance of initialization depends on two aspects: one is the data itself, including the degree of overlap, sample size, and dimension. The other is the allowable computation cost. Besides, the choice of hyperparameters for some algorithms also has a crucial impact on the final results.

In order to optimize the initial value of the EM algorithm and the estimated results, we propose a new iterative initialization method of EM algorithm for GMM under the situation with a known *K* in this paper. This method is to calculate the mean vector and covariance matrix of sample as the initial value of the iteration rather than to start with many different random initial conditions. Then, the optimal feature vector is selected from the candidate feature vectors by the maximum Mahalanobis distance as a new partition vector for clustering. The parameter values are renewed continuously according to the clustering results. To verify its applicability, we compared the proposed method with other two popular initialization methods on simulated and real datasets, respectively.

## 2 Materials and methods

### 2.1 EM algorithm for Gaussian mixture models

For *d*-dimensional random variable *X* with *n* samples, the probability distribution of a finite Gaussian mixture model can be expressed by a weighted sum of *K* components [22]:

$$\begin{array}{c} P\left(X|\Theta \right)=\sum_{m=1}^{K}\alpha_{m}p_{m}\left(X|\theta_{m}\right), \end{array}$$ (1)

where *α*_*m*_ is *m*-th mixing proportion, which must satisfy *α*_*m*_ > 0, *m* = 1, …, *K* and $\sum_{m=1}^{K}\alpha_{m}=1$. In Eq (1), *θ*_*m*_ = {*μ*_*m*_, Σ_*m*_} is the set of parameters of the *m*-th component, where *μ*_*m*_ and Σ_*m*_ denote the mean vector and covariance matrix of the *m*-th mixture component, respectively. *p*_*m*_ is the probability density function of the *m*-th component:

$$\begin{array}{c} p_{m}\left(X|\theta_{m}\right)=\frac{1}{{\left(2\pi \right)}^{d/2}{|\Sigma_{m}|}^{1/2}}\text{exp}\{\frac{1}{2}{\left(X-\mu_{m}\right)}^{T}\Sigma_{m}^{-1}\left(X-\mu_{m}\right)\}, \end{array}$$ (2)

where *d* is the dimension of the feature space and |⋅| denotes the determinant of a matrix. Thus, Θ = {*α*_1_, …, *α*_*m*_, *μ*_1_, Σ_1_, …, *μ*_*m*_, Σ_*m*_} is an unknown set of the parameters and it has to be estimated in the mixture learning process. The number of components *K* is either known or must be determined in the learning process. In this paper it is assumed that *K* is known.

Suppose *X* = {**x**_**1**_, **x**_**2**_, …, **x**_**n**_} is independent and identically distributed. For *K* = 1, Θ can be solved by MLE:

$$\begin{array}{c} \Theta_{MLE}=\text{arg}\;\underset{\Theta }{\text{max}}\left\{\text{log}\;L(\Theta )\right\}. \end{array}$$ (3)

Where *L*(Θ) refers to the likelihood function and is given by:

$$\begin{array}{c} L\left(\Theta \right)=\prod_{i=1}^{n}P(x_{i}|\Theta ). \end{array}$$ (4)

For *K* > 1, the solution of this maximization problem cannot be obtained in a closed form. As a numerical optimization method, EM algorithm is often used for such cases. EM algorithm is an iterative optimization strategy. Each iteration is divided into two steps called the expectation step (E-step) and the maximization step (M-step). For GMM, it can be divided into the following steps after giving the initial parameters Θ^(0)^ [23]:

• E-step: The posterior probability of the *s*-th observation belonging to the *K*-th component, *h*_*m*_(**x**_**i**_), is calculated by:

$$\begin{array}{c} h_{m}^{\left(s\right)}\left(x_{i}\right)=\frac{\alpha_{m}^{\left(s-1\right)}p_{m}\left(x_{i}|\theta_{m}^{\left(s-1\right)}\right)}{\sum_{j=1}^{K}\alpha_{j}^{\left(s-1\right)}p_{j}\left(x_{i}|\theta_{j}^{\left(s-1\right)}\right)}, \end{array}$$ (5)

where *i* = 1, 2, …, *n* and *m* = 1, 2, …, *K*. *s* = 1, 2, … represents the iteration number.

• M-step: Given the posterior probabilities $h_{m}^{\left(s\right)}\left(x_{i}\right)$, the updating formulas of parameter Θ^(*s*)^ are as follows:

$$\begin{array}{c} \alpha_{m}^{\left(s\right)}=\frac{1}{n}\sum_{i=1}^{n}h_{m}^{\left(s\right)}\left(x_{i}\right) \end{array}$$ (6)

$$\begin{array}{c} \mu_{m}^{\left(s\right)}=\frac{\sum_{i=1}^{n}h_{m}^{\left(s\right)}\left(x_{i}\right)*x_{i}}{\sum_{i=1}^{n}h_{m}^{\left(s\right)}\left(x_{i}\right)} \end{array}$$ (7)

$$\begin{array}{c} \Sigma_{m}^{\left(s\right)}=\frac{\sum_{i=1}^{n}h_{m}^{\left(s\right)}\left(x_{i}\right)\left(x_{i}-\mu_{m}^{\left(s\right)}\right){\left(x_{i}-\mu_{m}^{\left(s\right)}\right)}^{T}}{\sum_{i=1}^{n}h_{m}^{\left(s\right)}\left(x_{i}\right)}. \end{array}$$ (8)

The E-steps and M-steps are iterated until a convergence criterion is met. In this paper, A convergence criterion of relative improvement based on log likelihood is adopted:

$$\begin{array}{c} \frac{\text{log}P(X|\Theta^{\left(s\right)})-\text{log}P(X|\Theta^{\left(s-1\right)})}{\text{log}P(X|\Theta^{\left(s-1\right)})}<\varepsilon , \end{array}$$ (9)

where *ε* ≪ 1 was a pre-specified tolerance level (in this paper, *ε* = 1*e* − 5).

### 2.2 Initialization procedure

In this section, the proposed methodology (denoted as MRIPEM) is introduced under the premise that the number of components *K* is known. The initialization method of this paper involves the idea of clustering. Starting from the number of clusters is 1, the mean vectors, covariance matrices, and mixing proportions are gradually renewed in each iteration. The initialization procedure can be described in detail by the following steps:

1. Compute the population mean vector *μ*_1_ and covariance matrix Σ_1_ by MLE and set *m* = 2.
  $$\begin{array}{c} \mu_{1}=\frac{1}{n}\sum_{i=1}^{n}x_{i} \end{array}$$ (10)
  $$\begin{array}{c} \Sigma_{1}=\frac{1}{n}\sum_{i=1}^{n}\left(x_{i}-\mu_{1}\right){\left(x_{i}-\mu_{1}\right)}^{T}. \end{array}$$ (11)
2. Choose *t* (*t* is a parameter of the method) feature vectors randomly from *X* to form *X*_*l*_ = {**x**_**l1**_, **x**_**l2**_, …, **x**_**lt**_}. For each **x**_**li**_*ϵX*_*l*_, compute the minimal squared Mahalanobis distance [24] to the mean vectors *μ*_1_, *μ*_2_, …, *μ*_*m*−1_ that have already been chosen:
  $$\begin{array}{c} dmin^{2}\left(x_{li}\right)=\underset{j=1,\ldots ,m-1}{min}d_{m}^{2}\left(\mu_{j},\Sigma_{j},x_{li}\right), \end{array}$$ (12)
  where $d_{m}^{2}\left(\mu_{j},\Sigma_{j},x_{li}\right)$ is given by:
  $$\begin{array}{c} d_{m}^{2}\left(\mu_{j},\Sigma_{j},x_{li}\right)=\left(x_{li}-\mu_{j}\right){\left(\Sigma_{j}\right)}^{-1}{\left(x_{li}-\mu_{j}\right)}^{T}. \end{array}$$ (13)
3. Select $p_{m}=\text{arg}\;\underset{x_{li}\in X_{l}}{\text{max}}dmin^{2}\left(x_{li}\right)$ as the division standard of the *m*-th component. In this way, *p*_*m*_ and *μ*_1_, *μ*_2_, …, *μ*_*m*−1_ which have been calculated constitute *m* partition vectors.
4. Divide *X* into *m* partitions {*C*_1_, *C*_2_, …, *C*_*m*_} by using Euclidean distance, i.e. assign sample points **x**_**i**_ to the nearest partition vector.
5. According to the partition {*C*_1_, *C*_2_, …, *C*_*m*_} obtained in step 4, update the mean vectors, covariance matrices and mixing proportions of each component:
  $$\begin{array}{c} \mu_{j}=\frac{1}{|C_{j}|}\sum_{x_{i}\in C_{j}}x_{i} \end{array}$$ (14)
  $$\begin{array}{c} \Sigma_{j}=\frac{1}{|C_{j}|}\sum_{x_{i}\in C_{j}}\left(x_{i}-\mu_{j}\right){\left(x_{i}-\mu_{j}\right)}^{T} \end{array}$$ (15)
  $$\begin{array}{c} \pi_{j}=\frac{n}{|C_{j}|}, \end{array}$$ (16)
  where |⋆| represents the number of sample points in the ⋆.
6. If Σ_*j*_ is not positive definite, use a spherical covariance matrix instead:
  $$\begin{array}{c} \Sigma_{j}=\frac{1}{d|C_{j}|}\sum_{x_{i}\in C_{j}}∥x_{i}-\mu_{j}{∥}^{T}\cdot I_{D}, \end{array}$$ (17)
  where *I*_*D*_ denotes the *d*-dimensional identity matrix.
7. *m* = *m* + 1 if *m* < *K* then go to step 2. Otherwise, terminate the algorithm.
8. Set the manual loop parameter *r* that represents the number of runs of the initialization method and take the results corresponding to the best Adjusted Rand Index (ARI) [25] value as the output of the final initialization.

### 2.3 Properties of the initialization procedure

The iterative initialization method of this paper is a stochastic strategy, and it depends on samples completely. If *K* = 1, the initial parameter value is obtained in step 1 by using MLE. The algorithm iterates from step 2, gradually increasing the number of partitions until *X* is divided into *K* components.

The main idea of step 2 and step 3 is to use the minimal squared Mahalanobis distance to sift out the next optimal partition vector that is used for dividing the sample. When dividing the samples, our purpose is to make the selected partition vectors as far as possible from each other. This can reduce the probability of different components of samples being divided into the same component. In step 2, the *t* sample points are selected randomly from *X* as the candidate vectors. Then, the minimal squared Mahalanobis distance is calculated from the *t* candidate vectors to the determined *m* − 1 centers. The *m*-th partition vector is determined according to the maximum distance calculated above (Step 3). It should be noted that Mahalanobis distance is selected as the standard for determining the partition vector because it is an effective multivariate distance metric taking into account the relationship between various feature vectors [26]. In addition, the sensitivity study on the selection of *t* for different numbers of *K*(*K* = 2, 3, 4, 5, 7, 9) was made according to ARI value. In fact, ARI is an evaluation index proposed by Hubert and Arabie [25] to measure the clustering performance. The larger the ARI value, the more consistent the clustering results of the two partitions. When ARI takes a negative value, it indicates that the labels of the two partitions are independently distributed. When ARI is equal to 1, it indicates that the two partitions are identical.

**Fig 2:**
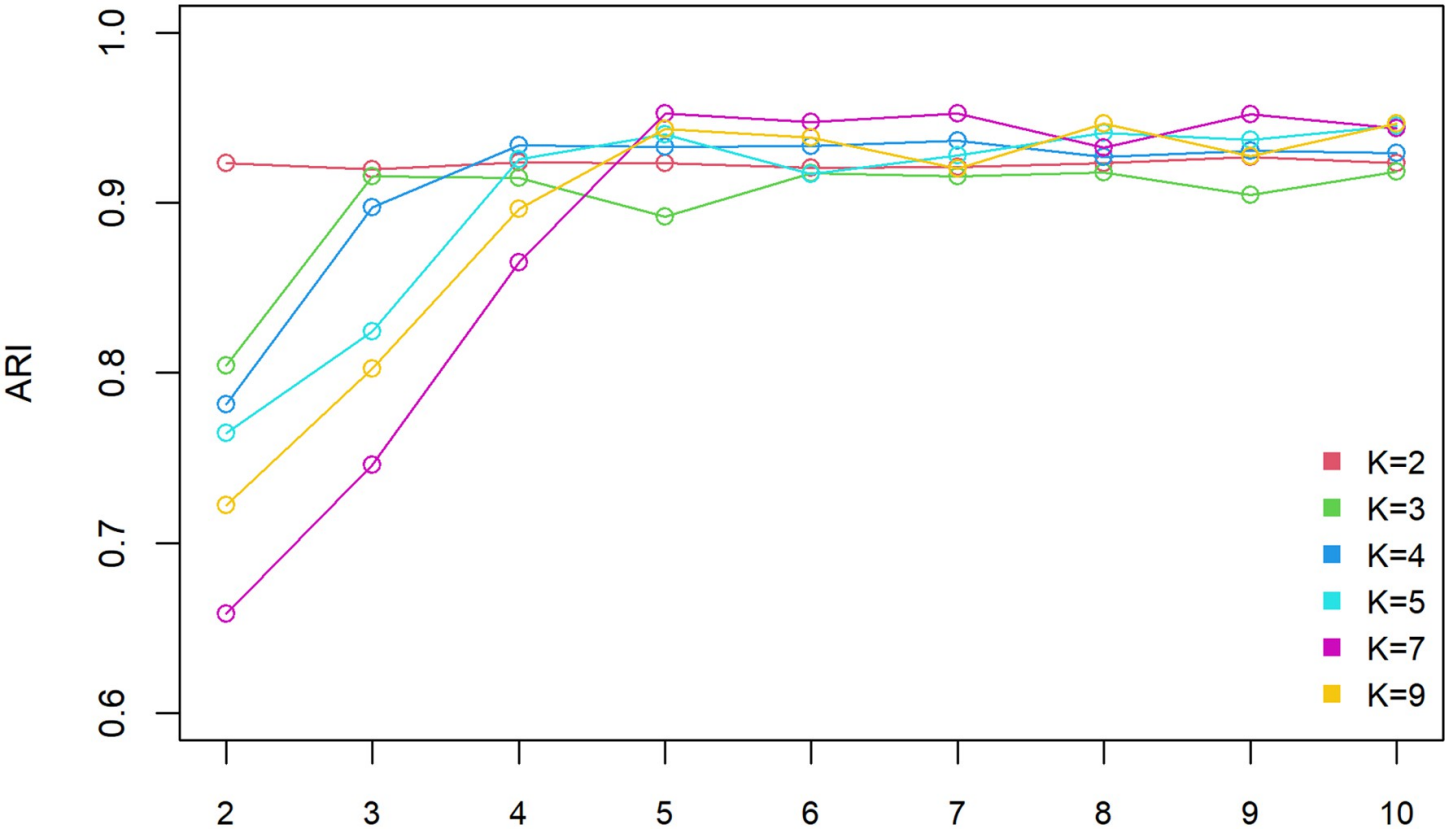
Determination of the best t. For each *K*, other parameters settings were fixed: loop parameter *r* = 1, number of samples per cluster *n*_*C*_ = 200, dimension *p* = 5. Considering the randomness of the algorithm, each group of experiments (*K*, *t*) was repeated 30 times. For each (*K*, *t*), the ARI value was determined using the ARI mean of 30 repeated experiments.

As can be seen from Fig 2, when *K* ⩽ 5 and *t* ⩽ *K*, the ARI value rise rapidly until *t* reaches *K*. When *t* > *K*, the ARI value gradually tends to a plateau. Therefore, we define *t* = *K* for *K* < 5. Similarly, when *K* > 5, the ARI value gradually grows with the increase of *t* until *t* = 5. The ARI value fluctuates little after *t* > 5. Therefore, we define *t* = 5 for *K* ≥ 5. The simulation experiments indicate that we also can get excellent results by bringing *t* = 5 (if *K* ≥ 5) sample points into step2 while the sample size is much larger than *t*. Choosing *t* instead of all *n* sample points means that the amount of iteration calculation will be greatly reduced, thus, the running speed of the algorithm is accelerated.

The sample points are clustered (Step 4) through the new partition vector (Step 3). The clustering results are used to estimate the *m*-th parameter values, which are mean vectors, covariance matrices, and mixing proportions. Step 5 lists the calculation formula of each parameter. In step 6, the covariance matrix is replaced by the spherical covariance matrix when it is not positive definite. This usually happens when the cluster is too small at the early stage of the iteration. The spherical covariance matrix is not only a positive definite matrix, but also can be constructed quickly without being affected by the iterative process. The parameter values are continuously updated during the process of iteration. The iteration stops and the last results are the outputs of the initialization method (Step 7) When *m* = *K*.

**Fig 3:**
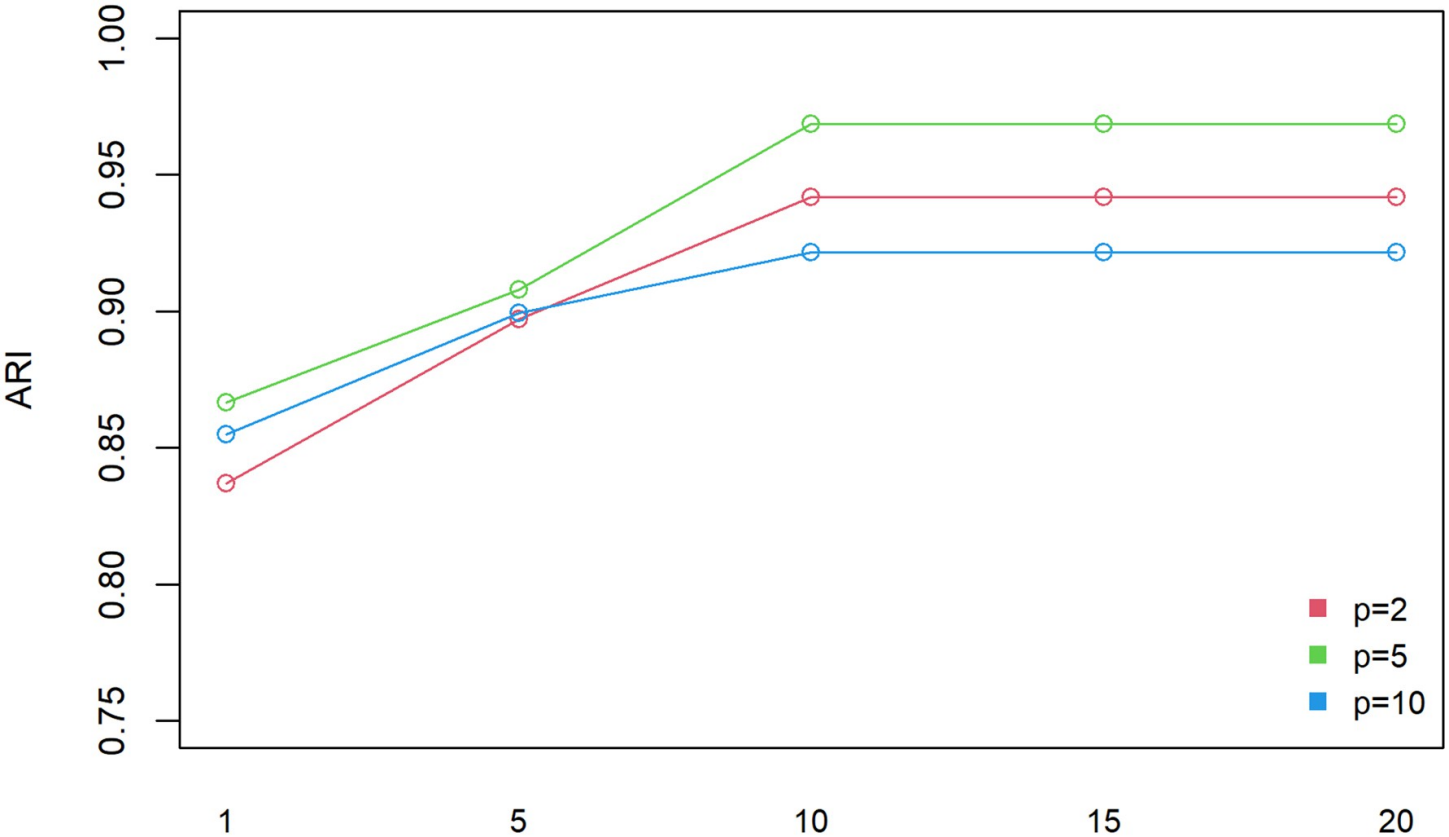
Determination of the best r. Other parameters settings: the number of components *K* = 20, sample size *n* = 4000.

Due to the random nature of the algorithm, the results will vary from run to run. In order to get the best initialization results, we have added a loop parameter *r* inside the initialization procedure representing the number of times to run our initialization method (Step 8). In *r* runs, ARI is a performance criterion to evaluate the results of each initialization. The initialization results corresponding to the maximum value of ARI is selected as the output of our initialization algorithm. Step 8 can be adjusted manually. Generally speaking, the greater the number of runs *r*, the better the initialization results. However, in order to improve the operation efficiency, we attempted to conduct a sensitivity analysis between the number of runs and the initialization results. The *r*(*r* = 1, 5, 10, 15, 20) experiments were conducted under three kinds of dimensions (*p* = 2, 5, 10), respectively. Considering the randomness of the algorithm, each group of experiments (*r*, *p*) was repeated 30 times and the ARI value was determined using the ARI mean of 30 repeated experiments. As show in Fig 3, the best performances are obtained under three kinds of dimensions when *r* = 10. Therefore, the manual loop parameter *r* is fixed to 10 in the subsequent experiments of this paper.

## 3 Results

In this section, the proposed initialization method is combined with the EM algorithm to estimate the parameters of the GMM through the clustering of the data, which is tested on the artificially generated datasets and real datasets, respectively. Meanwhile, our approach is compared with two other popular stochastic initialization strategies, which are emEM and RndEM.

The ARI value and the log-likelihood (log*L*(Θ)) were used to measure the performance of the data clustering. log*L*(Θ) is the objective function of the EM algorithm, and the larger it is, the better is. The combination of the two performance criteria can accurately measure the effectiveness of algorithms and make the experimental results more credible.

### 3.1 Simulation study

The artificial datasets for simulation study are generated by using the MixSim package in R [27], which can generate a finite mixture model with Gaussian components for prespecified levels of maximum pairwise overlap $\overset{ˇ}{\omega }$, average pairwise overlap $\bar{\omega }$, the number of clusters *K* and the dimension of samples *p*.

**Fig 4:**
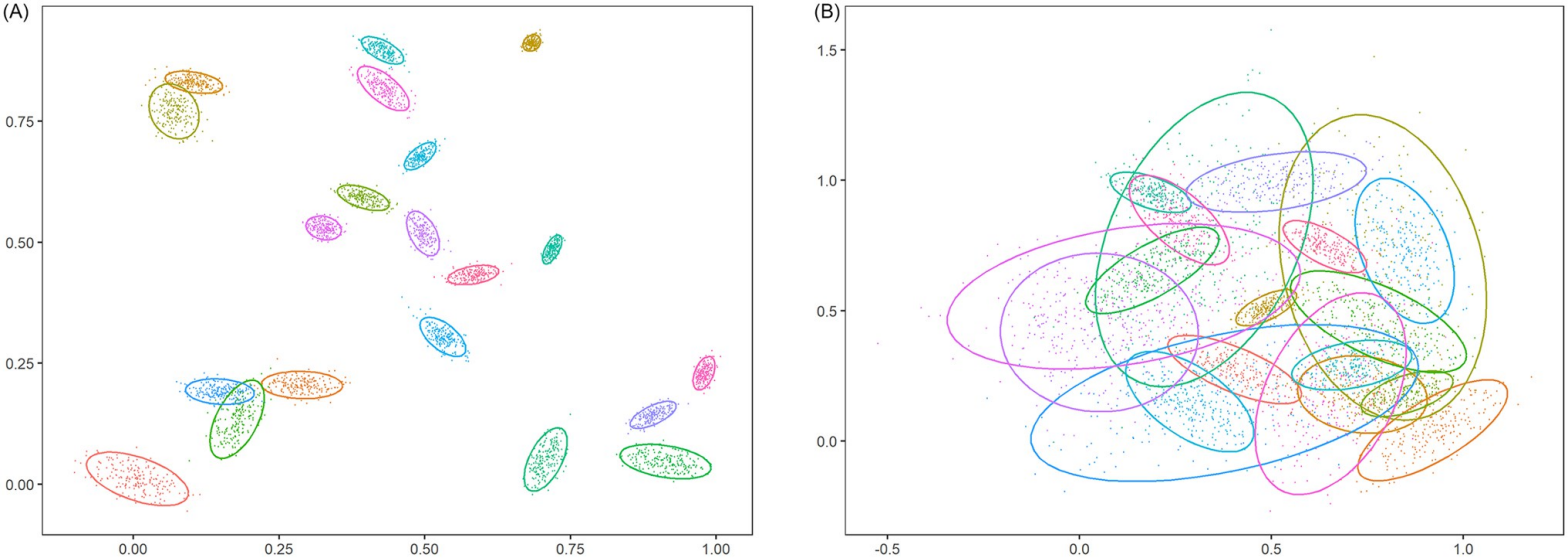
Two-dimensional datasets simulated from 10-component mixtures. A: $\bar{\omega }=0.001$ and $\overset{˘}{\omega }=0.04$. B: $\bar{\omega }=0.05$ and $\overset{˘}{\omega }=0.5$. The ellipses are centered around component means and represent 95% confidence regions.

Naturally, the dimensionality is considered to have an impact on the clustering performance of methods. The higher the dimension of the samples, the worse the clustering effect may be. Additionally, in Fig 4, both graphs A and B are made from the mixed Gaussian data generated by the generator (number of clusters: *K* = 10, dimension: *p* = 2, number of samples per cluster: *n*_*C*_ = 200). The difference is that the overlap degree of A ($\bar{\omega }=0.001$ and $\overset{˘}{\omega }=0.04$) is lower than the overlap degree of B ($\bar{\omega }=0.05$ and $\overset{˘}{\omega }=0.5$). As shown in Fig 4, the degree of data mixing will increase with the increase of $\bar{\omega }$. When $\bar{\omega }$ reaches 0.05, the degree of data mixing is quite serious. In this case, a very satisfactory result can not be obtained by any method. It can be concluded that the degree of overlap ($\bar{\omega }$ and $\overset{˘}{\omega }$) and the number of dimensions *p* are important parameters that affect the clustering results of the initialization methods.

Consequently, considering the different performances of the methods in different dimension *p*, average overlap $\bar{\omega }$ and maximum overlap $\overset{˘}{\omega }$, the number of components *K* is fixed to 20 and the total sample size *n* is fixed to 4000 (the number of samples *n*_*C*_ of each component is 200) in the simulation study. This experiment simulates three different dimensions (*p* ∈ {2, 5, 10}). Each dimension sets three values of average overlap ($\bar{\omega }\in \left\{0.0001,0.001,0.01\right\}$) and each average overlap sets two values of maximum overlap. In this way, a total of 18 experiments were carried out for different combination $\left(p,\bar{\omega },\overset{˘}{\omega }\right)$.

When three algorithms of emEM, RndEM and MRIPEM are compared, due to nature of stochastic strategies, we executed 30 different datasets for each triplet $\left(p,\bar{\omega },\overset{˘}{\omega }\right)$. In addition, to explore the stability of the results of 30 runs, we define var^*^:

$$\begin{array}{c} var^{*}=var\times 1000, \end{array}$$ (18)

where *var* represents the variance of ARI for 30 results. The reason for 1000 times magnification operation is that the range of ARI value is [0, 1], and the calculated variance value is too small to distinguish. Besides, there is a large difference in the value of the log likelihood function calculated from 30 different data, so it is meaningless to calculate its variance. Here we only calculate the variant of ARI variance (var^*^).

### 3.2 Results of simulation study

The comparison results of *p* = 2, *p* = 5 and *p* = 10 Gaussian mixture model datasets are listed in Tables 1–3 under three stochastic initialization methods, respectively. $\bar{I}$ and $\bar{L}$ represent the averages of ARI and log*L*(Θ) of 30 different datasets, respectively. *I*_*q*_ and *L*_*q*_ represent their corresponding third quartile. The values of ARI are accurate to 4 decimal places and the results of others are rounded up to 2 decimal places. The optimal value of each index has been highlighted in bold in the table.

**Table 1:** The comparison results of different $\bar{\omega }$ and $\overset{˘}{\omega }$ under three methods when *p* = 2.

Method	$\bar{\omega }$	0.0001		0.001		0.01	
	$\overset{˘}{\omega }$	0.01	0.015	0.1	0.15	0.3	0.4
MRIPEM	$\bar{I}$	**0.9730**	**0.9909**	**0.9537**	**0.9614**	**0.7777**	**0.8071**
	$\bar{L}$	**12889.66**	**13958.63**	**10068.74**	**12329.19**	**4754.87**	**3778.89**
	*I*_*q*_	**0.9980**	**0.9988**	**0.9792**	**0.9791**	**0.8161**	**0.8256**
	*L*_*q*_	**13616.60**	**14616.13**	**10915.82**	**13326.39**	**4935.85**	**3716.05**
	*var* ^*^	**1.02**	**0.39**	**0.76**	**0.55**	**0.68**	**0.46**
emEM	$\bar{I}$	0.8580	0.8355	0.8257	0.8584	0.7736	0.7414
	$\bar{L}$	12178.29	12882.32	9486.79	11767.31	4738.22	3679.89
	*I*_*q*_	0.8469	0.8832	0.8587	0.8838	0.7926	0.7543
	*L*_*q*_	13014.69	13976.94	10136.48	12900.92	4934.13	3584.34
	*var* ^*^	1.13	3.14	2.18	1.70	1.70	0.58
RndEM	$\bar{I}$	0.8933	0.8814	0.8535	0.8507	0.7119	0.7559
	$\bar{L}$	12326.64	13172.46	9673.00	11758.41	4650.84	3695.08
	*I*_*q*_	0.9225	0.9225	0.8802	0.8678	0.7381	0.7995
	*L*_*q*_	13173.78	14232.17	10165.81	13048.89	4799.56	3609.16
	*var* ^*^	1.04	2.54	2.40	1.36	0.94	2.07

Other parameter settings: *K* = 20, *n* = 4000, *t* = 5, *r* = 10.

**Table 2:** The comparison results of different $\bar{\omega }$ and $\overset{˘}{\omega }$ under three methods when *p* = 5.

Method	$\bar{\omega }$	0.0001		0.001		0.01	
	$\overset{˘}{\omega }$	0.01	0.015	0.1	0.15	0.3	0.4
MRIPEM	$\bar{I}$	**0.9863**	**0.9812**	**0.9612**	**0.9651**	**0.7839**	**0.8137**
	$\bar{L}$	**19603.89**	**22951.83**	**14441.47**	**16458.43**	**2536.95**	**3680.60**
	*I*_*q*_	**0.9983**	**0.9986**	**0.9818**	**0.9818**	0.7948	**0.8341**
	*L*_*q*_	**21850.09**	**25080.13**	**15168.96**	**17192.86**	2727.93	**4372.49**
	*var* ^*^	**0.57**	**0.76**	**0.65**	**0.68**	0.33	**0.41**
emEM	$\bar{I}$	0.8693	0.8294	0.8824	0.8857	0.7791	0.7966
	$\bar{L}$	19133.66	22498.65	14088.53	16131.29	2536.84	3490.30
	*I*_*q*_	0.8952	0.8899	0.9212	0.9166	**0.7969**	0.8211
	*L*_*q*_	21394.62	24686.68	14757.57	16837.87	**2762.54**	4330.93
	*var* ^*^	8.17	45.57	1.62	1.72	**0.31**	1.56
RndEM	$\bar{I}$	0.8739	0.8746	0.8467	0.8343	0.7361	0.7641
	$\bar{L}$	18967.73	22189.57	13926.75	15827.97	2429.54	3417.45
	*I*_*q*_	0.8907	0.9230	0.8729	0.8379	0.7724	0.7873
	*L*_*q*_	21227.24	24542.77	14834.54	16288.88	2747.39	4255.13
	*var* ^*^	2.08	2.36	0.88	0.71	2.33	1.14

Other parameter settings: *K* = 20, *n* = 4000, *t* = 5, *r* = 10.

**Table 3:** The comparison results of different $\bar{\omega }$ and $\overset{˘}{\omega }$ under three methods when *p* = 10.

Method	$\bar{\omega }$	0.0001		0.001		0.01	
	$\overset{˘}{\omega }$	0.01	0.015	0.1	0.15	0.3	0.4
MRIPEM	$\bar{I}$	**0.9756**	**0.9800**	**0.9405**	**0.9177**	0.6738	0.5907
	$\bar{L}$	**20043.87**	**23450.43**	**10902.43**	**14794.39**	-9597.66	-13373.93
	*I*_*q*_	**0.9974**	**0.9979**	**0.9646**	**0.9256**	0.6853	0.6334
	*L*_*q*_	**21915.60**	**25603.39**	**12208.31**	**15998.47**	-8279.14	-12860.28
	*var* ^*^	**0.79**	**0.76**	**0.74**	**1.09**	**0.37**	4.25
emEM	$\bar{I}$	0.9183	0.8946	0.8575	0.8998	**0.7449**	0.6715
	$\bar{L}$	19620.80	22901.37	10674.20	14746.22	**-8901.69**	**-12316.50**
	*I*_*q*_	0.9407	0.8890	0.9148	0.9199	**0.7649**	**0.7265**
	*L*_*q*_	21444.06	24965.52	12195.23	15907.85	**-7413.76**	**-11826.79**
	*var* ^*^	1.51	0.90	14.50	1.94	1.66	72.80
RndEM	$\bar{I}$	0.8716	0.8856	0.8787	0.8723	0.7180	**0.6827**
	$\bar{L}$	19352.43	22812.75	10527.50	14557.12	-8985.49	-12396.52
	*I*_*q*_	0.8832	0.8854	0.9076	0.8937	0.7369	0.7241
	*L*_*q*_	21335.29	24940.08	12136.25	15641.88	-7581.46	-12073.11
	*var* ^*^	0.99	1.18	1.19	1.78	1.35	**2.51**

Other parameter settings: *K* = 20, *n* = 4000, *t* = 5, *r* = 10.

Obviously, the three initialization methods perform differently with varying degrees of overlap and dimensionality. As show in Table 1, the values of five indexes indicate that MRIPEM is significantly better than emEM and RndEM for six different combinations of overlap. When $\bar{\omega }=0.0001$ and $\bar{\omega }=0.001$, the $\bar{I}$ value of emEM and RndEM are both below 0.9 and differ greatly from MRIPEM. When $\bar{\omega }=0.01$, although the difference of $\bar{I}$ values among the three algorithms is reduced, the method of MRIPEM is still the best. Particularly, when *w* = 0.0001, the *I*_*q*_ value of MRIPEM is close to 1. This displays that more than 75% of the results in 30 repeated experiments trials are consistent with the real classification results. Besides, the var^*^ value of MRIPEM is the smallest compared with the other two algorithms, indicating that the obtained results of MRIPEM are relatively stable. The results of Table 2 illustrate that the situation of *p* = 5 is similar to that *p* = 2. In terms of the averages of ARI and log*L*(Θ), MRIPEM is also better than the other two methods for six overlap combinations. For the corresponding third quartile, when $\bar{\omega }=0.01$ and $\overset{˘}{\omega }=0.3$, the result of MRIPEM is slightly smaller than that of emEM. However, the difference of *I*_*q*_ is only 0.26% and the difference of *L*_*q*_ is only 1.27%. In Table 3, when the dimension is increased to 10, the results of MRIPEM are still best for $\bar{\omega }=\left\{0.0001,0.001\right\}$. It’s a pity that MRIPEM provides the worst results compared to the other two methods when $\bar{\omega }=0.01$. Nevertheless, the MRIPEM method still remains relatively stable to a certain extent from the results of var^*^.

In a word, the comparisons of ARI reveal that the MRIPEM method has a higher accuracy and can reduce the probability of data being wrongly divided. The comparisons of log-likelihood function values show that the parameters calculated by the MRIPEM method have high fitting validity. According to the values of var^*^, the MRIPEM method is relatively stable and has little volatility compared with the other two initialization methods. In addition, it can be found that the three stochastic initialization methods have different degrees of decline with the increase of the degree of overlap and dimension. This reflects from another aspect that the degree of data overlap and dimension are the important reasons that affect the performance of the algorithm.

**Fig 5:**
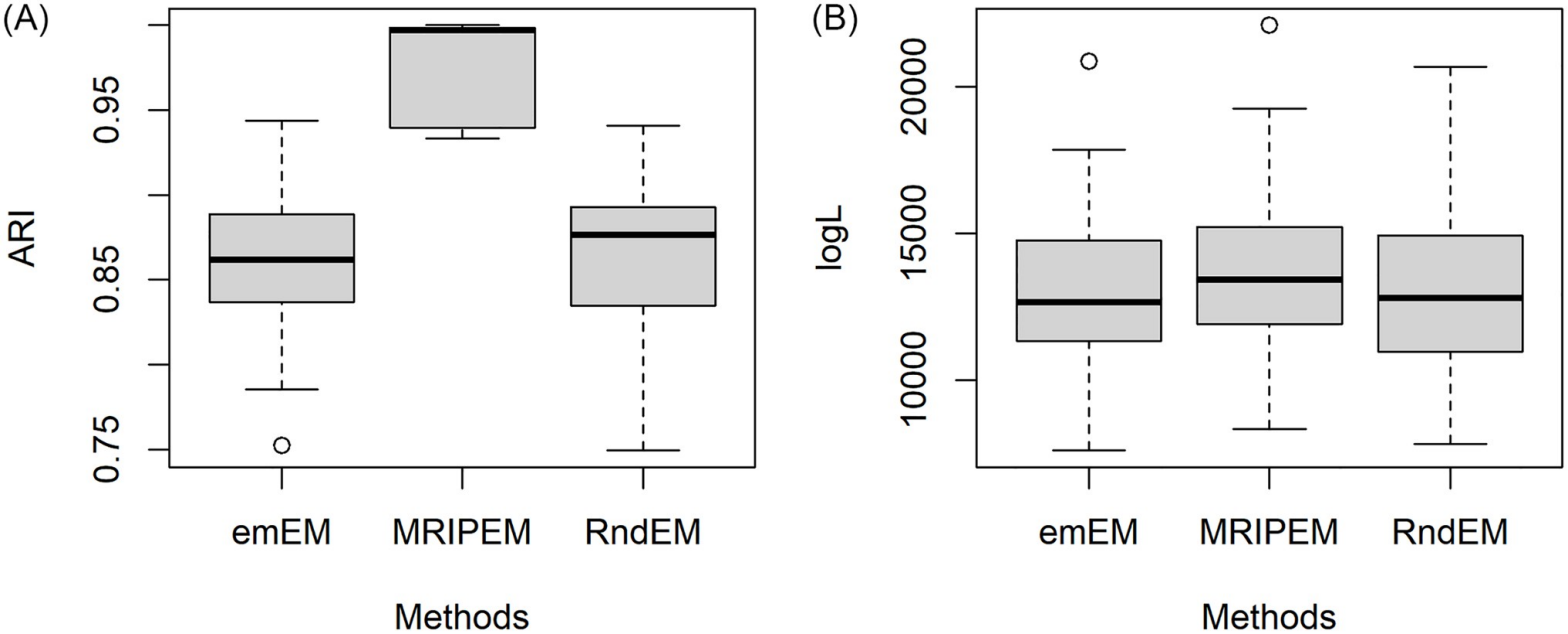
Boxplots of the ARI and log*L*(Θ) for the emEM, RndEM and the proposed algorithm MRIPEM. A: Comparison of the ARI for three methods. B: Comparison of the log*L*(Θ) for three methods. Each boxplot is constructed based on 30 different datasets with $\left\{p,\bar{\omega },\overset{˘}{\omega }\right\}=\{2,0.0001,0.01\}$.

**Fig 6:**
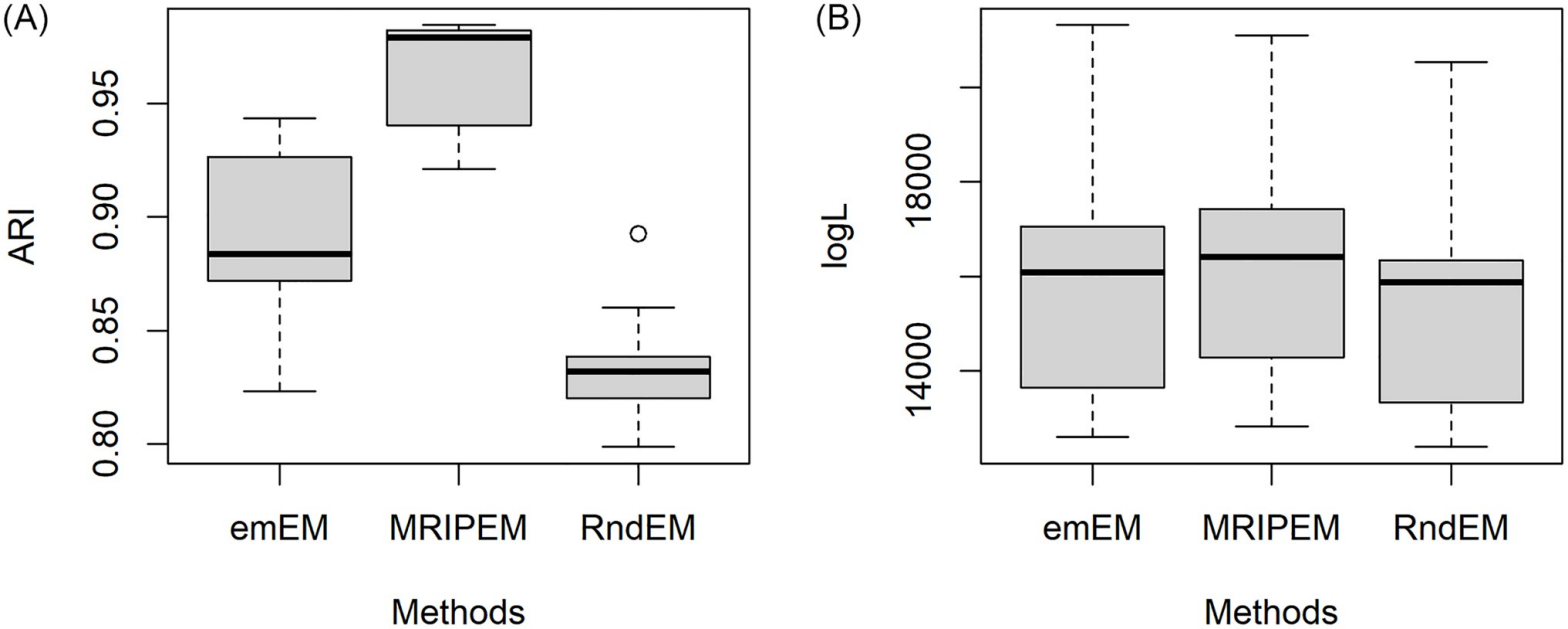
Boxplots of the ARI and log*L*(Θ) for the emEM, RndEM and the proposed algorithm MRIPEM. A: Comparison of the ARI for three methods. B: Comparison of the log*L*(Θ) for three methods. Each boxplot is constructed based on 30 different datasets with $\left\{p,\bar{\omega },\overset{˘}{\omega }\right\}=\{5,0.001,0.15\}$.

**Fig 7:**
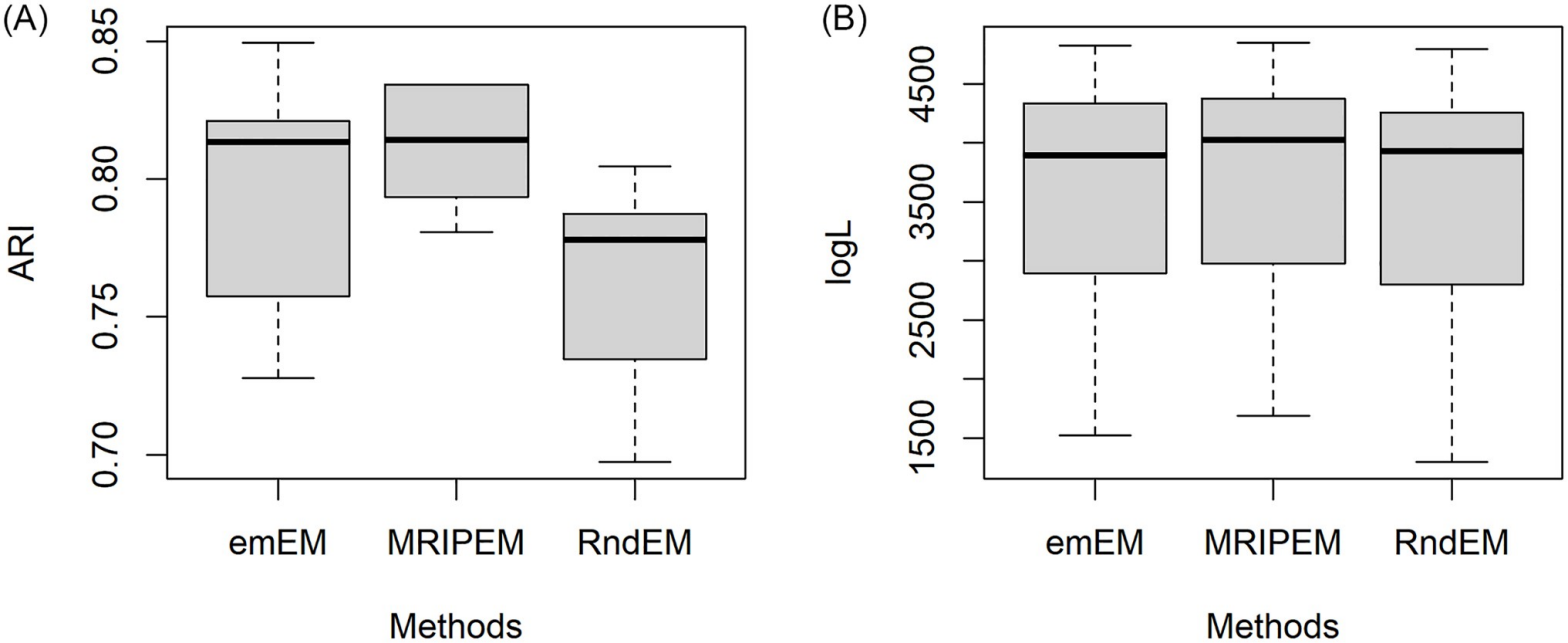
Boxplots of the ARI and log*L*(Θ) for the emEM, RndEM and the proposed algorithm MRIPEM. A: Comparison of the ARI for three methods. B: Comparison of the log*L*(Θ) for three methods. Each boxplot is constructed based on 30 different datasets with $\left\{p,\bar{\omega },\overset{˘}{\omega }\right\}=\{5,0.01,0.4\}$.

**Fig 8:**
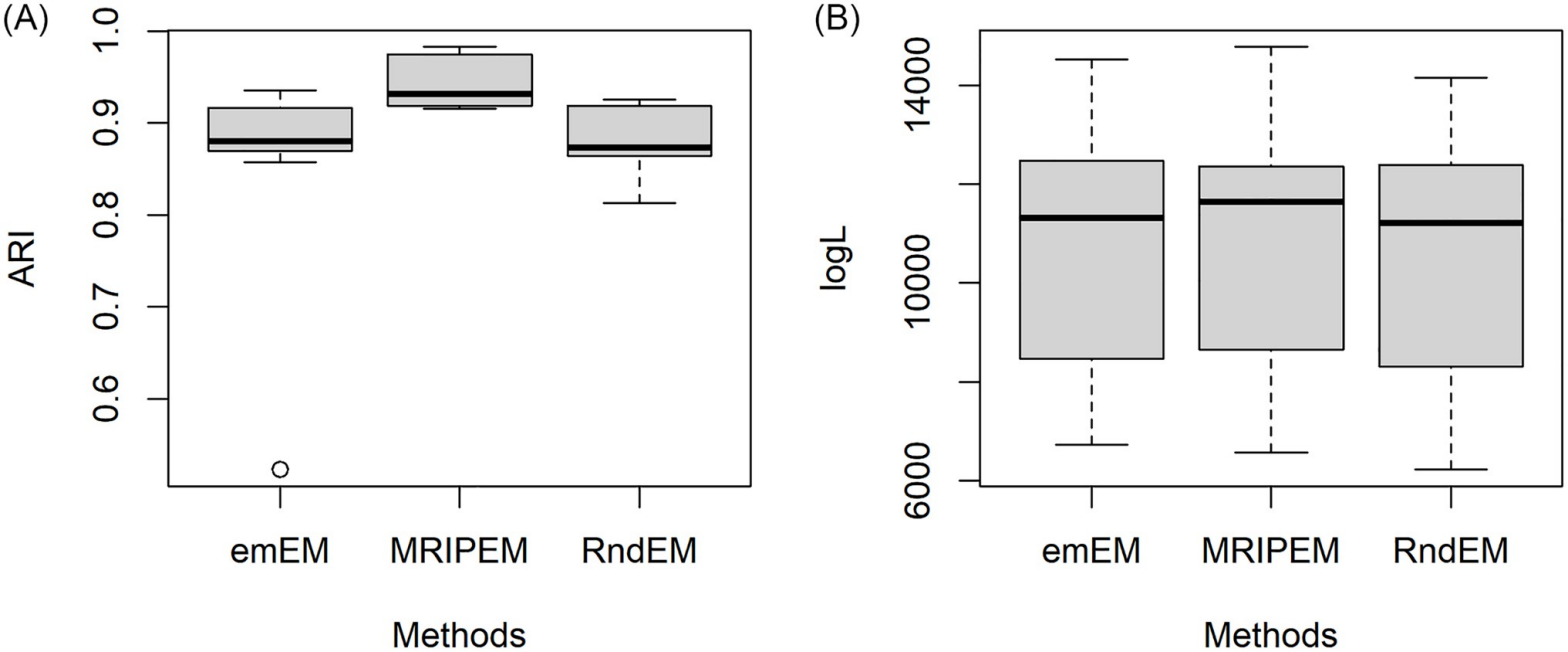
Boxplots of the ARI and log*L*(Θ) for the emEM, RndEM and the proposed algorithm MRIPEM. A: Comparison of the ARI for three methods. B: Comparison of the log*L*(Θ) for three methods. Each boxplot is constructed based on 30 different datasets with $\left\{p,\bar{\omega },\overset{˘}{\omega }\right\}=\{10,0.001,0.1\}$.

In order to better present the specific information for results of the 30 datasets, we extract four representative groups from the 18 triplets, and draw the boxplots of ARI and log*L*(Θ) in Figs 5–8, respectively. In Fig 5, we draw with the settings of the least overlap ($\bar{\omega }=0.0001$) and *p* = 2. Fig 6 represents the medium overlap ($\bar{\omega }=0.001$) and *p* = 5. From Figs 5 and 6, it can be found that the MRIPEM is better than the other two methods in both ARI and log*L*(Θ). The middle line of the box is the median of the data, representing the average level of sample. Actually, the values of MRIPEM in ARI are far ahead of the other two methods and its median is close to 1. Fig 7 is drawn with the settings of the high degree of overlap ($\bar{\omega }=0.01$) and *p* = 5, and Fig 8 represents the medium degree of overlap ($\bar{\omega }=0.001$) and *p* = 10. Figs 7 and 8 indicate that the MRIPEM method continues to excel in whatever its respect in accordance with medians of ARI and log*L*(Θ). From the closeness of the upper and lower limits of boxes, the results of MRIPEM fluctuate less.

On the premise of fixing the total number of samples *n* and the number of clusters *K*, the results of the simulation experiments show that MRIPEM is significantly better than the other two classic algorithms when the degrees of overlap($\bar{\omega }=\left\{0.0001,0.001\right\}$) are not high. The MRIPEM method achieves the highest results on both ARI and log*L*(Θ). The Adjusted Rand Index is close to 1, almost perfect classification. In fact, the result of MRIPEM even appeared several times with ARI = 1 during the process of simulation.

For the $\left\{p,\bar{\omega }\right\}=\left\{10,0.01\right\}$, the MRIPEM method is inferior to the other two methods. Maybe the high degree of overlap and dimensionality increase the probability of selecting partition vectors from the same component by using maximum Mahalanobis distance. Therefore, it seems that our initialization algorithm is suitable for well-separated and low-dimensional samples. In fact, the performance of all three algorithms will decrease in varying degrees with the increase of overlap and dimension. It’s obvious that more overlapping data increases the difficulty of distinguishing them.

### 3.3 Real datasets study

In this section, we use datasets of four known class labels from UCI machine learning database [28] and KEEL-dataset repository [29] to demonstrate the validity of the proposed method, namely *Seeds*, *Aff*, *Appendicitis*, and *SKM*. These datasets vary from dimension of feature space, sample size, number of classes, and degree of overlap.

1. *Seeds* dataset
  The *Seeds* dataset is a commonly used classification experimental dataset. The dataset contains 210 instances, which belong to three different varieties of wheat: Kama, Rosa, and Canadian. There are 70 instances in each category, and each instance contains 7 attributes: area *A*, perimeter *P*, compactness *C*, length of kernel, width of kernel, asymmetry coefficient, and length of kernel groove. According to the seven attributes, which variety each seed belongs to can be predicted.
2. *Aff* dataset
  The full name of *Aff* dataset is Algerian forest fires. It includes 244 instances that regroup data of two regions of Algeria, namely the Bejaia region located in the northeast of Algeria and the Sidi Bel-abbes region located in the northwest of Algeria, with 122 instances for each region. Through 7 attributes, 244 examples were divided into “fire” (138 classes) and “not fire” (106 classes).
3. *Appendicitis* dataset
  The data represents 7 medical measures taken over 106 patients on which the class label represents if the patient has appendicitis (class label 1) or not (class label 0).
4. *SKM* dataset
  It is the real dataset about the students’ knowledge status about the subject of Electrical DC Machines. In accordance to five attributes including the degree of study time for the goal object materials (STG), it can be divided the knowledge level of students.

The above datasets all have known their respective dimension sizes, but the degrees of overlap are unknown. To get the level of clustering complexity for an existing classification dataset, the average pairwise overlap and maximum pairwise overlap of four real datasets were calculated by using the *overlap* function of MixSim package in R [27]. The information of four real datasets is listed in Table 4 as follows:

**Table 4:** Information on real datasets.

**Datasets**	*n*	*p*	*k*	$\bar{\omega }$	$\overset{˘}{\omega }$
*Seeds*	210	7	3	0.0080	0.0172
*Aff*	244	7	2	0.0075	0.0075
*Appendicitis*	106	7	2	0.1977	0.1977
*SKM*	403	5	4	0.0202	0.0607

Parameter elucidation: *n*, *p*, *k*, $\bar{\omega }$, $\overset{˘}{\omega }$ represent the number of samples, the number of features, the number of clusters, the average pairwise overlap and the maximum pairwise overlap, respectively.

### 3.4 Results of real datasets study

As in the simulation experiments, considering that the three initialization methods are all stochastic strategies, this experiment was repeated 30 times and each time was started with a different seed of the random number generator. The results of three initialization methods under four real datasets are displayed in Table 5.

**Table 5:** Comparative results of ARI and log*L*(Θ) of three stochastic initialization methods on four real datasets.

Method	*Seeds*		*Aff*		*Appendicitis*		*SKM*	
	ARI	log*L*(Θ)	ARI	log*L*(Θ)	ARI	log*L*(Θ)	ARI	log*L*(Θ)
MRIPEM	**0.7882**	1250.71	**0.7104**	**4364.10**	**0.3787**	**1162.58**	**0.3347**	**234.15**
emEM	0.6299	1276.66	0.6157	4257.49	0.2227	1157.14	0.2051	231.69
RndEM	0.5869	**1281.56**	0.6554	4267.09	0.2227	1157.14	0.2676	233.02

**Fig 9:**
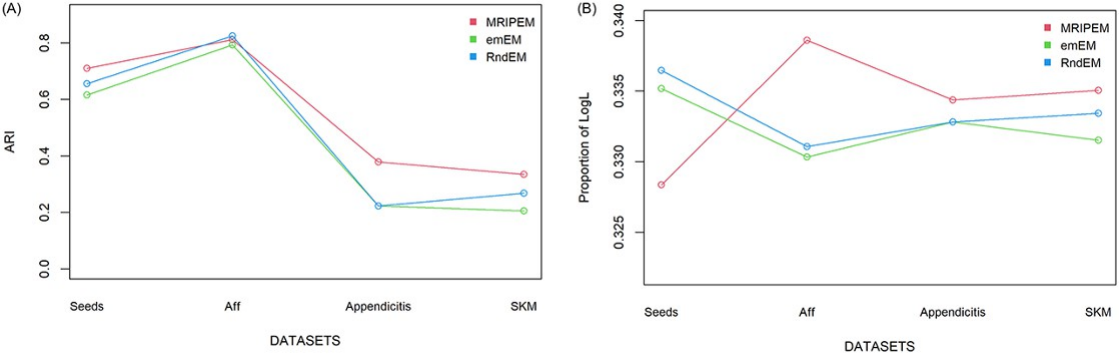
Line charts of ARI and log*L*(Θ) values of three methods under four real datasets. A: Comparison of the ARI for three methods. B: Comparison of the log*L*(Θ) for three methods.

Fig 9 intuitively shows the difference of ARI and log*L*(Θ) values of the three methods under the four real datasets. Due to the large difference between the log*L*(Θ) values of different datasets, the log*L*(Θ) results is converted into a proportional form (For each real dataset, log*L*(Θ) of each method / the sum of log*L*(Θ) of three methods).

It can be seen from Table 5 and Fig 9 that the ARI value of the MRIPEM is the largest among the three methods in four kinds of datasets. In terms of log*L*(Θ), MRIPEM is the largest except on the *Seeds* dataset. These results demonstrate that the classification results of MRIPEM algorithm are closer to those of real datasets. In more detail, the MRIPEM method is improved by 25.13% and 8.39% respectively compared with the second-ranked method on *Seeds* and *Aff* datasets in terms of ARI. However, the MRIPEM method is only 2.41% worse than the first ranked RndEM method on *Seeds* datasets in the aspect of log*L*(Θ). From the overall results, the value of log*L*(Θ) has little difference under the three algorithms. The lower ARI value of the last two datasets may be caused by the relatively high degree of overlap. Even so, MRIPEM is still ahead of emEM and RndEM whether on ARI or log*L*(Θ).

## 4 Discussion and conclusion

In this paper, a novel iterative initialization method of the EM algorithm for the estimation of the parameters of GMM was proposed. This new method, named MRIPEM, incorporates the ideas of multiple restarts, iterations, and clustering. The performance of MRIPEM was assessed by ARI and log*L*(Θ) indexes, and compared with the other two popular initialization strategies on simulated datasets and real datasets, respectively.

In fact, the initial mean and covariance matrix of the iteration of the MRIPEM algorithm are calculated by taking all the sample data as one component, which is different from other algorithms to randomly generate the initial mean and variance. According to the Mahalanobis distance, the candidate vector farthest from all the determined center vector points is taken as the partition vector. In this way, the greater the distance, the greater the probability of being selected as the partition vector, otherwise the probability is smaller. That is, the probability of proper initialization of all component centers increases. After clustering the sample data according to the partition vector, the mean and variance of each component are continuously updated in each iteration. Obviously, the MRIPEM algorithm completely depends on the sample dataset itself, which reduces the randomness to a certain extent. In addition, the setting of the manually adjustable loop parameter *r* can also reduce the randomness to a certain extent and improve the accuracy of the results. The comparison results of simulation datasets and real datasets under the three algorithms confirm that the MRIPEM algorithm shows excellent accuracy and robustness in low dimensions and low overlaps. Therefore, the MRIPEM is recommended to apply to the well-separated datasets of the low dimension or after dimensionality reduction.

However, it is a well-known fact that no single method can outperform the others in all cases, and our algorithm is no exception. Similar to other initialization methods, the performance of the MRIPEM method also decreases when the degree of overlap and the dimensionality are both high. Maybe the high dimension and high overlap will increase the probability of misclassification of partition vectors determined by the maximum Mahalanobis distance. Nevertheless, on the whole, the MRIPEM method can still be regarded as a promising algorithm in terms of situations in low-dimension and low-overlap.

### 4.1 Statements

The real datasets that support the findings of this study are openly available in UCI machine learning database at https://archive.ics.uci.edu/ml and KEEL-dataset repository at https://sci2s.ugr.es/keel/datasets.php (Ref [28, 29]).
